# Chronic Inflammatory Demyelinating Polyradiculoneuropathy with Diplopia Caused by an Alternative Coronavirus Disease 2019 Vaccine

**DOI:** 10.1155/2024/8584482

**Published:** 2024-06-11

**Authors:** Satoshi Saito, Mutsumi Iijima, Misa Seki, Ayato Shimomura, Kazuo Kitagawa

**Affiliations:** Department of Neurology, Tokyo Women's Medical University School of Medicine, 8-1 Kawada-cho Shinjuku-ku, Tokyo, Japan

## Abstract

The etiology of chronic inflammatory demyelinating polyradiculoneuropathy (CIDP) remains elusive and is believed to involve multiple contributing factors. There have been cases linking CIDP to the coronavirus disease 2019 (COVID-19) mRNA vaccine. However, there are no documented instances following alternative vaccines. We report a case of a 48-year-old woman, previously vaccinated with Pfizer-BioNTech's COVID-19 vaccine (BNT162b2), who subsequently received the Moderna mRNA-1273 vaccine. Within 2 days postvaccination, she developed diplopia and numbness in the lower limbs' distal extremities. Cerebrospinal fluid analysis exhibited protein-cell dissociation, while F-wave studies showed demyelinating activity in the bilateral tibial nerves. Given the disease's progressive nature, the patient was presumed to have CIDP and commenced steroid pulse therapy and intravenous immunoglobulin therapy. The onset of CIDP may be associated with variations in mRNA sequences and vaccine constituents.

## 1. Introduction

Before the COVID-19 (coronavirus disease 2019) pandemic, common vaccines included live, inactivated, toxoid, and recombinant protein types. The advent of mRNA and viral vector vaccines has proven effective against COVID-19, with Pfizer-BioNTech's BNT162b2 and Moderna's mRNA-1273 considered leading mRNA vaccines. These vaccines deliver the genetic instructions for the viral spike protein, prompting cellular production of this protein and subsequent immune response [1]. Neurological adverse events such as myelitis, acute disseminated encephalomyelitis, and Guillain–Barré syndrome have been observed, potentially caused by molecular mimicry between microbial and host antigens [2]. Nevertheless, whether the association between the COVID-19 vaccine and autoimmune manifestations is coincidental or causal remains unresolved.

Chronic inflammatory demyelinating polyradiculoneuropathy (CIDP) manifests as chronic, often relapsing-remitting muscle weakness and sensory deficits in both distal and proximal limb muscles, persisting for over two months. The etiology of CIDP remains undefined, with various humoral and cellular immune responses implicated, yet without identifying specific autoantibodies or pathogenic antigens. The 2021 European Academy of Neurology/Peripheral Nerve Society guidelines reclassify atypical CIDP as CIDP variants, detailing distinct diagnostic and differential approaches [3]. Distal CIDP, more symptomatic in the lower limbs, and cases featuring cranial neurological manifestations such as diplopia have been documented [4].

This report describes a CIDP case with diplopia following administration of the mRNA-1273 vaccine, which fulfills the criteria for possible distal CIDP. Notably, the patient had no adverse reactions to three prior doses of BNT162b2.

## 2. Case Presentation

A 48-year-old woman received her fourth COVID-19 vaccination with mRNA-1273, having been previously inoculated thrice with BNT162b2. Two days postvaccination, she developed diplopia, which was followed a month later by a decline in muscle strength in both lower limbs, necessitating stair handrail assistance. After six months, numbness affected the soles and toes bilaterally, and by eight months, her diplopia had intensified.

Her physical examination revealed a height of 157 cm, normal pupils with rapid light reflexes, mild entropion in the left eye, and slightly limited bilateral eye abduction; however, convergence was intact. Diplopia occurred in all gaze directions. Manual muscle testing indicated normal strength in the upper limbs but reduced strength in lower limb muscles, including the iliopsoas (5/4+), quadriceps (5/5), and others. Biceps, triceps, and flexor tendon reflexes were normal; however, patellar and Achilles tendon reflexes were absent. She reported numbness below both ankles despite normal superficial sensation. Vibration sensation diminished up to the tibial tubercle, but position sensation remained unaffected. The Romberg test was positive, and she required handrail support for balance.

**Figure 1:**
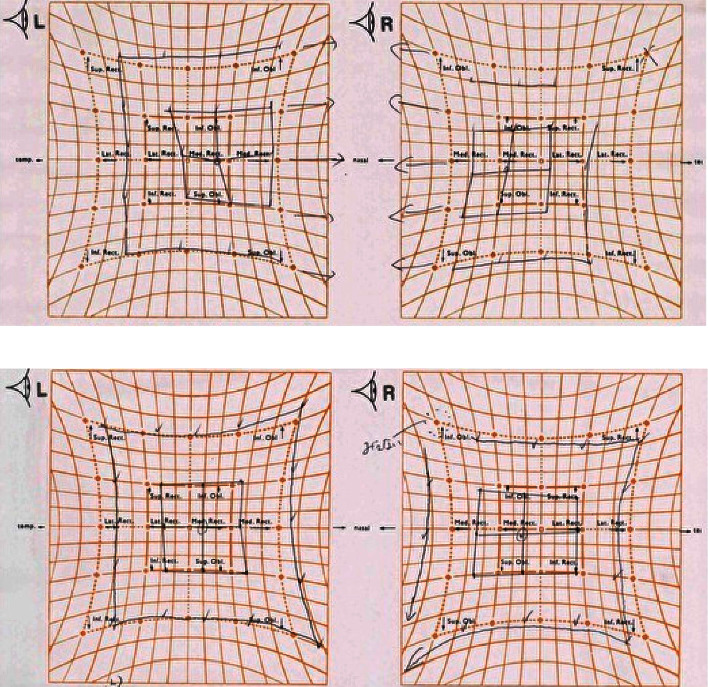
Results of the Hess screen test: (a) restricted eye movement during abduction before intravenous immunoglobulin therapy. (b) Improved eye movement during abduction after intravenous immunoglobulin therapy.

**Figure 2:**
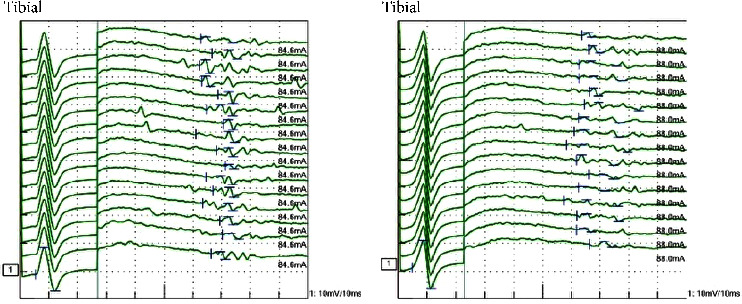
The F-wave study in the left (a) and right (b) tibial nerve: bilateral latency dispersion expansions indicate demyelination.

Laboratory tests showed normal blood counts, organ function, electrolytes, and HbA1c at 5.9%. Tests for syphilis, hepatitis B, and C were negative. Inflammatory markers were within normal ranges, and vitamin levels varied, with VitB12 at 283 pg/mL and VitB1 at 34 ng/mL. Immunoglobulin levels were IgG 1,365 mg/dL, IgA 193 mg/dL, IgM 103 mg/dL, and M protein was negative. Autoantibodies such as antinuclear, anti-CCP, anti-SSA, and anti-SSB antibodies were negative. The cerebrospinal fluid analysis showed white blood cells at 2.3/*μ*L, red blood cells at 0.0/*μ*L, protein at 75 mg/dL, IgG index 0.48, and negative oligoclonal bands. Bilateral abduction defects were noted on the Hess red-green test (Figure 1(a)). Additional diagnostic tests, including ice pack, Tensilon, and repetitive nerve stimulation, were negative, and nerve conduction studies revealed no significant abnormalities. F-wave studies indicated bilateral tibial nerve involvement (Figure 2) with a prolonged minimum latency (Table 1). In a previous report, the normal minimum latency of the F-wave at a height of 157 cm was 42 ± 5 ms (mean ± standard deviation) [5]. In our case, the minimum latency was 57.5 ms at the right tibial and 61.0 ms at the left tibial, indicating a prolongation of more than 20% from the standard deviation on both sides.

The clinical presentation of deep sensory disturbances, muscle weakness, and absent tendon reflexes in the lower limbs pointed to polyneuropathy. Following the EAN/PNS diagnostic criteria, the F-wave findings and cerebrospinal fluid analysis led to a diagnosis of possible distal CIDP, likely immune-mediated and potentially vaccine-induced. Steroid pulse therapy improved symptoms in the lower limbs from day four, and by day 15, lower limb tendon reflexes had also improved, though diplopia persisted. Following immunoglobulin therapy, diplopia symptoms diminished, and Hess test results ameliorated by day 11 of treatment (Figure 1(b)).

## 3. Discussion

The BNT162b2 (Pfizer-BioNTech) and mRNA-1273 (Moderna) vaccines contain mRNA sequences encoding the SARS-CoV-2 spike protein encapsulated within lipid nanoparticles. The dosage differs, with BNT162b2 containing 30 µg and mRNA-1273 containing 100 µg of mRNA [6]. Although mRNA-1273 has been associated with delayed skin reactions, commonly referred to as “Moderna arm,” these typically resolve within several days [7, 8]. A comparative study of individuals receiving BNT162b2 for their first two doses and either BNT162b2 or mRNA-1273 for their third dose indicated significantly higher anti-spike IgG and neutralizing antibody titers with mRNA-1273 [9], suggesting a superior preventative effect due to its higher mRNA content [10]. Moreover, mRNA-1273 uses the ionizable lipid SM-102 to achieve a stable nanoparticle structure, whereas BNT162b2 chooses a different lipid named ALC-0315 [11]. These raise the possibility that differences in composition or mRNA dosage may influence the onset of neurological diseases, as observed in the current case.

Although the mechanism by which the COVID-19 vaccine causes autoimmune disease is unknown, autoimmunity in the case of the influenza, hepatitis B, and human papillomavirus vaccines may be induced by molecular mimicry [12]. Molecular mimicry is one of the leading mechanisms by which infectious or chemical agents may induce autoimmunity. It occurs when similarities between foreign and self-peptides favor an activation of autoreactive T or B cells by a foreign-derived antigen in a susceptible individual. Other factors such as breach in central tolerance, nonspecific bystander activation, or persistent antigenic stimuli may also contribute to the development of autoimmune diseases [13]. In addition, adjuvants are highly heterogeneous chemical compounds, with immune response stimulation as their common property. In impaired tolerogenicity, exposure to these similar elements could induce autoimmunity [14]. A similar mechanism may be involved in the development of autoimmune diseases by the COVID-19 vaccine, but further studies are needed.

The patient's condition progressed chronologically and did not align with the typical Guillain–Barré or Fisher syndromes; it also tested negative for antiglycolipid antibodies. Differentiation from other potential causes of neuropathy, such as anti-MAG IgM neuropathy, diabetic neuropathy, hereditary neuropathy, POEMS syndrome, and vasculitic neuropathy, was achieved, with all tests returning negative [4]. In our case, the time to onset was relatively early, occurring 2 days after vaccination. Several similar cases have been reported, with Guillain–Barré syndrome occurring 2 days after the second dose of mRNA-1273 [15], transverse myelitis occurring 3 days after the first dose of BNT162b2 dose [16], and encephalitis occurring 1 day after the first BNT162b2 dose [17]. Furthermore, our case did not align with the typical CIDP, as it was accompanied with diplopia. While cranial neuropathies are reported in various percentages among CIDP types, with facial and bulbar palsy occurring in 9% and ocular motor impairment in 5%, this case's ocular motility disorder was concluded to be a consequence of CIDP [5].

CIDP is categorized as an immune-mediated neuropathy with documented associations with the COVID-19 mRNA vaccines [18, 19]. Guillain–Barré syndrome postvaccination incidence rates are 3.29 per million doses for Janssen's Ad26.COV2.S, 0.29 per million for BNT162b2, and 0.35 per million for mRNA-1273 [20]. When comparing the incidence rates of Guillain–Barré syndrome following COVID-19 vaccines to other vaccines, the rate is significantly higher for COVID-19 vaccines at 49.7 per 10 million doses, compared to 0.19 for the influenza vaccine and 0.16 for other vaccines. However, the incidence associated with COVID-19 vaccines falls within the expected range for Guillain–Barré syndrome in the general population [21]. Although our case implies an association with the COVID-19 vaccine, current evidence does not conclusively establish that COVID-19 mRNA vaccines promote immune-mediated neuropathies.

BNT162b2 and mRNA-1273 are mRNA vaccines that facilitate the production of the SARS-CoV-2 spike protein. Differences in their mRNA quantities and components may potentially contribute to the development of chronic progressive polyneuropathies, exemplified by conditions such as CIDP accompanied by diplopia.

## Figures and Tables

**Table 1:** Nerve conduction and the F-wave study.

(Nerve conduction study)							
Motor nerve	Side	Stimulation site	Amplitude (mV)	Latency (ms)	Velocity (m/s)		

Median	Left	Wrist	20.5	4.5			
		Elbow	19.7	9.0	46.7		
Ulnar	Left	Wrist	15.3	2.8			
		Elbow − 2.5	14.4	7.0	48.2		
		Elbow + 2.5	13.9	7.9	52.6		
Peroneal	Left	Ankle	7.8	4.2			
		Popliteal	5.7	11.8	46.4		
Tibial	Left	Ankle	15.6	5.2			
		Popliteal	10.8	13.8	40.9		

Sensory nerve	Side	Stimulation site	Amplitude (*μ*V)	Latency (ms)	Velocity (m/s)		

Median	Left	Wrist	15.0	5.5	41.4		
Ulnar	Left	Wrist	10.4	4.5	51.5		
Sural	Left	Sural	24.7	4.7	42.0		

(F-wave study)							
Nerve	Side	Stimulation site	Amplitude (*μ*V)	Latency (ms)	Velocity (m/s)	Occurrence (%)	Prolongation^∗^ (%)

Median	Right	Wrist	30–250	29.4–30.8	54.4–57.6	81	
	Left		60–750	28.9–32.8	50.7–59.2	94	

Tibial	Right	Ankle	60–190	57.5–66.3	28.6–34.1	100	122
	Left		80–250	61.0–71.5	31.5–36.8	100	129

^∗^Prolongation indicates the percentage of the minimum latency in comparison with the normal value (47 ms).

## Data Availability

The data are available from the first author upon request.
